# Higher Levels of Serum Uric Acid Have a Significant Association with Lower Incidence of Lower Urinary Tract Symptoms in Healthy Korean Men

**DOI:** 10.3390/metabo12070649

**Published:** 2022-07-14

**Authors:** Jiwon Hwang, Seungho Ryu, Joong Kyong Ahn

**Affiliations:** 1 Division of Rheumatology, Department of Internal Medicine, Samsung Changwon Hospital, Sungkyunkwan University School of Medicine, 158 Paryong-ro, Masanhoewon-gu, Changwon 51353, Korea; ninedw@empas.com; 2 Department of Occupational and Environmental Medicine, Kangbuk Samsung Hospital, Sungkyunkwan University School of Medicine, Samsung Main Building B2, 67 Sejong-daero, Jung-gu, Seoul 04514, Korea; 3 Division of Rheumatology, Department of Internal Medicine, Kangbuk Samsung Hospital, Sungkyunkwan University School of Medicine, 29 Saemunan-ro, Jongno-gu, Seoul 03181, Korea

**Keywords:** lower urinary tract symptoms, men, uric acid

## Abstract

Gout has been correlated with the risk of incident benign prostatic hyperplasia. In line with increasing prevalence of hyperuricemia, the aim of this study was to investigate the relationship between serum uric acid (SUA) level and the incidence of lower urinary tract symptoms (LUTS) among clinically healthy middle-aged men. We performed a cohort study in 101,091 Korean men without LUTS at baseline who completed health checkups between 2011 and 2016. LUTS were evaluated using the International Prostate Symptom Score, where a score ≥ 8 was defined as significant LUTS. Men were divided into six groups according to their SUA levels in mg/dL (<5.5, 5.5–6.4, 6.5–7.4, 7.5–8.4, 8.5–9.4, and ≥9.5). Throughout the follow-up—encompassing a total of 358,982.6 person years—13,424 people had significant LUTS (37.3 per 1000 person years for incidence rate). The multivariable models demonstrated that the highest level of SUA (≥9.5 mg/dL) was related to the lowest risk of significant LUTS compared with the reference category (<5.5 mg/dL) (0.77 (95% CI 0.59–0.99) for adjusted HR). In this large cohort composed of middle-aged men, higher SUA levels were related to a reduced risk of LUTS. This result suggests another potential role of SUA in restraining LUTS. Additional studies are needed to explain the underlying biological mechanisms of this phenomenological relationship.

## 1. Introduction

Lower urinary tract symptoms (LUTS) become more common as men age. LUTS are broadly grouped into three categories: hesitancy, poor and/or intermittent stream, straining, and dribbling as voiding symptoms; frequency, urgency, nocturia, and leaking as storage symptoms; and prolonged micturition and feeling of incomplete bladder emptying as post-micturition symptoms [1]. Roughly three-quarters of men reported at least one symptom that occurs ”sometimes”, while approximately half of men had at least one symptom “often” [2]. In Korea, the prevalence of LUTS in men ≥40 years old was as high as 70.6%, with 44.7% suffering from moderate to severe symptoms [3].

LUTS have negative influences on the quality of life and mental health, such as lower workplace productivity and higher levels of anxiety and depression [4,5]. Historically, prostate enlargement and subsequent bladder outlet obstruction were thought to explain the pathophysiology of LUTS [6]. The relationship between LUTS, prostate volume, and urodynamic parameters was investigated, but no causative relationship was identified between the parameters of prostatic hyperplasia and symptom severity. This suggests that other factors are involved in the development of LUTS [7]. Previous studies have shown the associations of urological and nonurological conditions: erectile dysfunction [8]; irritable bowel syndrome [9]; metabolic syndrome [10]; increased free thyroxine level [11]; and environmental stress [12].

Uric acid (UA) has recently been brought to the fore in the modulation of prostate cells. Depending on its chemical environment, UA can be either an antioxidant or pro-oxidant. Oxidative stress was considered to promote benign prostate hyperplasia (BPH) through activation of the inflammasome in response to UA [13]. Meanwhile, one population-based study demonstrated that uric acid lowering treatment (ULT) was associated with lower use of BPH medications and less frequent diagnosis of LUTS than in those who were not treated with ULT [14]. Another group found that ULT reduced prostate cancer cell growth by 37% [15].

Regardless of whether UA has a role in prostate pathology, the relationship between UA and LUTS remains unclear. Therefore, the objective of this study is to evaluate the relationship between serum uric acid (SUA) levels and the occurrence of LUTS in a large Korean sample composed of middle-aged men without LUTS at baseline.

## 2. Results

### 2.1. Characteristics of the Study Population

Table 1 displays the baseline characteristics of the 101,091 men in accordance with their SUA levels. The mean ± SD age was 38.1 ± 6.8 y, and the mean ± SD body mass index (BMI) was 24.5 ± 3.0 kg/m^2^. The average level of SUA was 6.17 mg/dL. Hyperuricemia was detected in 23.8% of participants. Compared to those with lower SUA levels, male participants with higher SUA levels were more likely: to be younger; to be more obese; to consume larger quantities alcohol; to have a history of hypertension with more severe systemic and diastolic blood pressure (BP); and to have higher total cholesterol, low-density lipoprotein (LDL) cholesterol, triglycerides, homeostasis model of assessment of insulin resistance (HOMA-IR), and high-sensitivity C-reactive protein (hsCRP). In contrast, a history of diabetes, current smoking, and high-density lipoprotein (HDL) cholesterol levels were negatively correlated with increasing SUA levels. The total caloric intake had no significant association with the SUA levels.

### 2.2. Relationship between SUA Levels and the Incidence of Significant LUTS

Throughout the follow-up of a total of 358,982.6 person years, 13,424 people had significant LUTS (37.3 per 1000 person years for incidence rate).

Table 2 demonstrates the relationship between the SUA levels and the development of significant LUTS. Sequential adjustment was performed in multivariable models. In a basic model adjusted for age, increasing SUA levels were negatively associated with the risk of significant LUTS (*p* for trend < 0.001). After additional adjustment for confounding factors, the corresponding HR of the highest category of SUA (≥9.5 mg/dL) was 0.74 (95% CI 0.58–0.93) for significant LUTS compared with the reference category (<5.5 mg/dL). There was a significantly negative linear trend across the SUA categories (*p* for trend < 0.001). The final multivariable model was adjusted for the estimated glomerular filtration rate (eGFR), total cholesterol, HOMA-IR, and hsCRP. In this final model, the HR in the highest SUA category was 0.77 (95% CI 0.59–0.99). However, the significant linear trend across the SUA categories was lost. After further exclusion of subjects who had newly detected diabetes and metabolic syndrome in their health checkup, analysis was carried out again, and the results were similar to the primary analysis: the highest category of SUA ≥9.5 mg/dL) was associated with the lowest risk of significant LUTS compared with the reference category (<5.5 mg/dL) (Supplementary Table S1).

**Figure 1:**
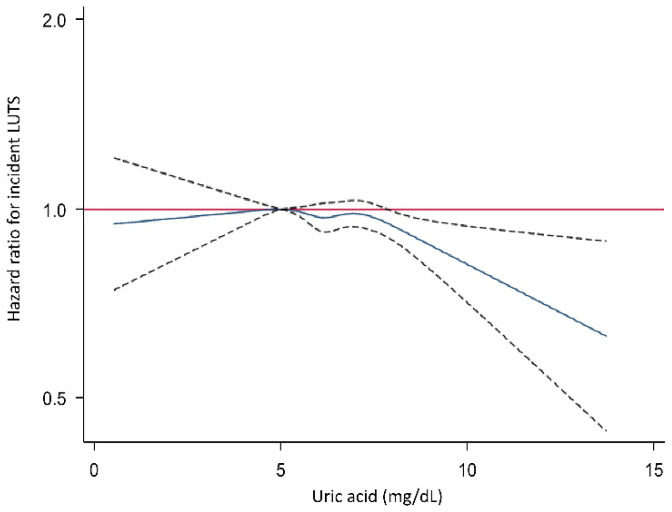
The relationship between serum uric acid levels and the incidence of significant LUTS. Curves were estimated from a Cox proportional hazards regression model representing the relationship between serum uric acid levels and incidence of significant LUTS. Broken lines represent 95% confidence intervals. LUTS, lower urinary tract symptoms.

The incidence rate of significant LUTS seemed to gradually decrease as the SUA level increased (Figure 1).

### 2.3. Modifying Effect of Age, Smoking, Alcohol Intake, Physical Activity, and BMI

The relationship between the SUA levels and the risk of significant LUTS was re-examined by pre-set subgroups (Table 3). The decreasing risk of significant LUTS with increasing SUA levels was most dominant in men <50 years of age. The HR in the highest SUA category was 0.65 (95% CI 0.50–0.84).

## 3. Discussion

In this large cohort of Korean men, higher SUA levels were negatively associated with a risk of significant LUTS. These patterns of association were similar in sequentially adjusted models. This association was predominant in men <50 years of age when the stratified analyses were performed with pre-set subgroups. To the best of our knowledge, this study is the first to demonstrate a relationship between SUA and risk of significant LUTS development, independent of physical activity, caloric intake, obesity, diabetes, hypertension, and comorbid conditions. This result, therefore, suggests that SUA might potentially have an effect on the severity of LUTS.

Unexpectedly, higher SUA levels were not associated with total caloric intake in our study population. This may be explained by Korean dietary habits, which may differ from Western diets with regard to the proportion of macronutrients in the overall caloric intake. In contrast, higher SUA levels were significantly associated with obesity, high blood pressure, alcohol consumption, lipid profiles, and insulin resistance. Dietary differences may contribute to variable urine pH despite a hyperuricemic state, which may explain the reduced incidence of LUTS [16].

Another intriguing observation was the modifying effect of age. In the stratified analyses by age subgroups, there was a stronger association between higher SUA levels and lower risk of LUTS in men <50 years than there was in those ≥50 years old. Therefore, the protective effect of higher SUA (against significant LUTS) appeared to decline after 50 years. Considering that aging is related to metaflammation underpinning inflammaging [17], it would make sense that SUA changes have variable effects according to a patient’s age and comorbid conditions. Elderly men ≥50 years old are also more likely to have comorbid conditions (related to aging) that may contribute to LUTS development. These age-related comorbidities may be less common in younger men; therefore, the effect of SUA seems more prominent in men <50 years old.

The pertinent mechanisms underlying the biological role of SUA in LUTS remain unclear. However, there are several potential explanations based on previous investigations. Previous studies mainly investigated prostate pathology related to hyperuricemia or gout. Men undergoing ULT had a lower risk of BPH than those without ULT [14]. This result is potentially explained by oxidative stress associated with hyperuricemia and the antioxidant effects of ULT. Another group found that in patients with chronic prostatitis, the urate concentration in prostatic secretions was proportionally associated with palpable pain [18]. Higher concentrations of urate and urine reflux were thought to play a part in crystal or stone formation inside the prostate gland, as well as serving as a foundation for bacterial prostatitis. Purine metabolites were also thought to serve as key metabolites in chronic inflammatory reactions. Considering the provocative relationship between the urate and prostate, changes in SUA level could (directly or indirectly) affect LUTS via the prostate.

One recent study demonstrated that changes in plasma urate levels influenced prostate cancer cell growth [15]. The extracellular effects of UA are thought to be mediated by the UA transporter, glucose transporter 9 (GLUT9). GLUT9 was found to be expressed significantly less in primary prostate cancer cell lines compared to normal prostate epithelial cells. This finding suggests that intracellular UA homeostasis is involved in prostate disease. Through immunohistochemistry on the various tissue arrays of prostate disease, GLUT9 expression was observed (in varying degrees) in normal tissue, BPH, and prostatitis other than prostate cancer. GLUT9 is a novel, facilitative glucose transporter that also has a unique specificity for UA [19]. The prostate, kidney, articular cartilage, and brain also express GLUT9. GLUT9 plays an important role in these tissues as a high-capacity urate transporter. Certain genetic polymorphisms have been associated with higher levels of SUA in gout and renal calculi [20]. In the human brain, the apical side of epithelial cells in the choroid plexus and the cilia of ependymal cells are known to the localized site of GLUT9. These results reflect the important role of urate in the central nervous system [21]. Considering the above results, we believe that urate via GLUT9 may work similarly in the development of LUTS.

Higher SUA levels are not always harmful. Hyperuricemia had a significantly positive correlation with pulmonary function [22] and bone mineral density in the lumbar spine of male patients [23]. It seems that the same level of higher SUA has opposite effects depending on tissue type and patient age. In this context, ULT in asymptomatic hyperuricemic subjects must be guided by specific evidence.

Despite our novel finding, this study has several limitations. First, the use of self-reported questionnaires increased the risk of recall bias. The questionnaires were not specified for family anamnesis regarding LUTS or other kidney/prostate disease. Secondly, testosterone levels were not routinely obtained in the health checkup program and therefore were unavailable for analysis. Instead, we attempted to minimize this bias by excluding men who were being medically treated for alopecia because this kind of medication can influence prostate growth. We also included men with a mean age of 38 years with preserved sexual function. Finally, as the present study focused on middle-aged men, our findings may not be generalizable to elderly men or to women. Thus, some of the mechanisms elaborated above might only be relevant to younger men.

## 4. Materials and Methods

### 4.1. Participants and Design

**Figure 2:**
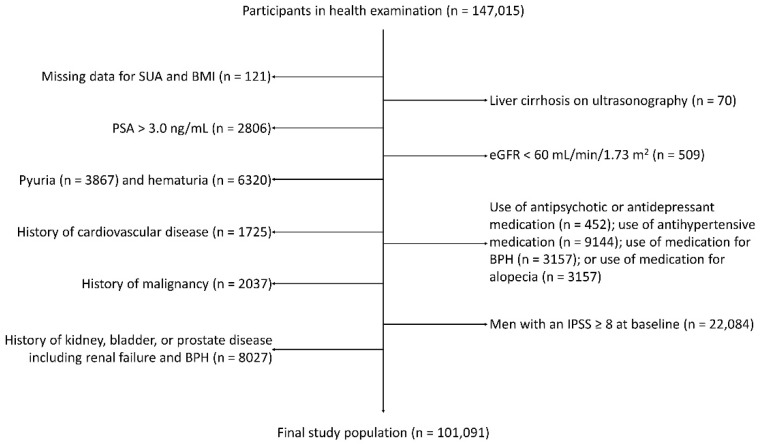
Selection of the study population. BMI, body mass index; BPH, benign prostate hyperplasia; eGFR, estimated glomerular filtration rate; IPSS, International Prostate Symptom Score; PSA, prostate specific antigen; SUA, serum uric acid.

The study population comprised adult men who participated in the health checkup program between January 2011 and December 2016 at Kangbuk Samsung Hospital Total Healthcare Centers in Seoul and Suwon, Korea. In total, 147,015 men who completed extensive questionnaires, including physical activity and the International Prostate Symptom Score (IPSS), and visited at least once for a follow-up by December 2017 were considered for inclusion. Exclusion criteria at baseline were as follows: missing data for SUA or BMI (n = 121); eGFR < 60 (mL/min/1.73 m^2^) (n = 509); serum prostate specific antigen (PSA) level ≥ 3 ng/dL (n = 2806); pyuria (n = 3867); hematuria (n = 6320); history of cardiovascular disease (n = 1725); history of malignancy (n = 2037); history of kidney, bladder, or prostate disease, including kidney stone, renal failure, and BPH (n = 8027); liver cirrhosis on ultrasonography (n = 70); use of antipsychotics or antidepressants (n = 452); use of medication for BPH (n = 3157); or use of medication for alopecia (n = 3157). Men with an IPSS ≥ 8 at baseline were also excluded (n = 22,084). Ultimately, 101,091 men were included (Figure 2). Among them, none had a history of gout or was on medication for gout.

### 4.2. Data Collection and Measurements

All the participants completed self-administered, standardized questionnaires at each visit. As previously described, the questionnaires collected demographics, education level, medical history, medication(s), smoking status, alcohol consumption, daily caloric intake, and physical activity [23,24]. Based on smoking history, subjects were categorized as current, former, and never smokers. Alcohol consumption was categorized as follows: none, moderate (<20 g/day), and high (≥20 g/day). Physical activity was classified as inactive, minimally active (600 MET minutes per week), and health-enhancing physically active (HEPA; 3000 MET minutes per week). Physical activity was assessed by the validated Korean version of the International Physical Activity Questionnaire Short Form [25].

Experienced nurses measured anthropometric items, including height, weight, and blood pressure (BP). The formula for BMI was kg/m^2^: weight (kg) divided by the squared height (m^2^). Obesity was defined as a BMI ≥ 25 kg/m^2^, which is the standard for Asian populations [26]. Hypertension was defined as a systolic BP ≥ 140 mmHg, diastolic BP ≥ 90 mmHg, or current treatment with antihypertensive medications.

Fasting samples were drawn in the morning to measure the serum concentrations of glucose, total cholesterol, LDL cholesterol, HDL cholesterol, triglycerides, hsCRP, and PSA [22,27]. The eGFR formula was from the Chronic Kidney Disease Epidemiology Collaboration equation [28]. HOMA-IR was used to estimate insulin resistance, which was calculated as the fasting insulin (mU/L) × fasting glucose (mmol/L)/22.5. The definition of diabetes was a fasting glucose concentration ≥ 126 mg/dL, glycated hemoglobin concentration ≥ 6.5%, or current treatment with antidiabetic medications or insulin. Hyperuricemia in men was defined as SUA ≥ 7 mg/dL.

The IPSS questionnaire is the most commonly used tool in the evaluation of LUTS severity. We used the validated Korean version of the IPSS, which employs seven questions regarding the following urinary symptoms: incomplete emptying, frequency, intermittency, urgency, weak stream, straining, and nocturia [29]. The answers are assigned points from 0 to 5. The total score ranges from 0 to 35, which is currently categorized as follows: asymptomatic or mild (0–7), and moderate or severe (≥8). We defined clinically significant LUTS as an IPSS score ≥ 8.

### 4.3. Statistical Analysis

The SUA levels (mg/dL) were divided into the following six groups: <5.5, 5.5–6.4, 6.5–7.4, 7.5–8.4, 8.5–9.4, and ≥9.5. Continuous variables are shown as means with standard deviations or medians with interquartile ranges. Categorical variables are shown as numbers and percentages.

The individual participants were followed from the baseline checkup either until the development of LUTS or the last checkup performed prior to 31 December 2017, whichever came first. The outcome variable was the development of clinically significant LUTS. The incidence rate was calculated as the number of incident cases divided by the person years of follow-up.

The relationship between the SUA levels and the risk of clinically significant LUTS was modeled using Cox proportional hazards regression to assess the hazard ratios (HRs) and 95% confidence intervals (CIs). Three models were generated with progressive adjustment. All of the models were adjusted for age in years. Multivariable model 1 was additionally adjusted for study center (Seoul, Suwon), year of checkup, education background (≤high school graduate or less, community college or university graduate, ≥graduate school, and unknown), smoking status (never, former, current, or unknown), alcohol consumption (0, <20, ≥20 g/day, or unknown), total caloric intake (in quintiles or unknown), physical activity (inactive, minimally active, HEPA, or unknown), BMI, and history of diabetes and hypertension. Multivariable model 2 was further adjusted for eGFR, total cholesterol, HOMA-IR, and hsCRP. In order to test the linear trend across categories, the ordinal variable was entered into each regression model.

We conducted stratified analyses in the pre-set subgroups: age (<50 vs. ≥50 y), smoking (none vs. ever), alcohol consumption (<20 g/day vs. ≥20 g/day), physical activity (HEPA; no vs. yes), and BMI (<25 kg/m^2^ vs. ≥25 kg/m^2^). Each stratum was analyzed using the final model. The interactions between the subgroups were tested using likelihood ratio tests to compare models with and without multiplicative interaction terms. All significance tests were two tailed. The statistical significance was established at *p* < 0.05. Data analyses were performed using STATA v14.2 (Stata Corp., College Station, TX, USA).

## 5. Conclusions

These results suggest that higher SUA levels can be associated with the severity of LUTS in healthy middle-aged men. This may indicate the potential role of SUA in the prevention of significant LUTS. Although this association seems to be favorable, the well-known negative effect of hyperuricemia on general health is not desirable. Further investigations are needed to elucidate the pertinent biological mechanisms of this association.

## Figures and Tables

**Table 1:** Baseline characteristics by uric acid level.

Characteristics	Overall	Uric Acid Level (mg/dL)						*p* for Trend
		<5.5	5.5–6.4	6.5–7.4	7.5–8.4	8.5–9.4	≥9.5	
Number	101,091	26,926	35,153	25,230	10,105	2809	868	
Age, y	38.1 ± 6.8	39.2 ± 7.3	38.1 ± 6.7	37.5 ± 6.4	36.9 ± 6.1	36.6 ± 5.8	36.1 ± 5.7	<0.001
Obesity (%)	39.6	27.6	35.3	46.7	58.5	67.3	74.4	<0.001
Current smoker (%)	36.7	37.5	36.5	36.6	36.5	35.0	33.1	0.001
Alcohol intake (%) ^1^	33.5	31.7	32.3	35.0	37.2	38.4	41.5	<0.001
HEPA (%)	16.3	17.6	16.2	15.7	15.3	15.3	17.3	<0.001
High education level (%) ^2^	89.8	88.3	90.0	90.7	90.6	90.0	89.0	<0.001
History of diabetes (%)	1.9	3.3	1.6	1.2	1.0	0.9	0.5	<0.001
History of hypertension (%)	5.0	4.0	4.5	5.4	7.1	9.0	10.7	<0.001
BMI, kg/m^2^	24.5 ± 3.0	23.6 ± 2.7	24.2 ± 2.7	25.0 ± 2.9	25.8 ± 3.0	26.6 ± 3.3	27.3 ± 3.4	<0.001
Systolic BP, mmHg	114.3 ± 11.4	112.5 ± 11.2	113.6 ± 11.1	115.3 ± 11.3	117.0 ± 11.8	118.8 ± 11.8	121.1 ± 12.3	<0.001
Diastolic BP, mmHg	73.6 ± 9.4	72.3 ± 9.1	73.1 ± 9.2	74.4 ± 9.4	75.6 ± 9.7	77.1 ± 9.6	78.8 ± 10.4	<0.001
Glucose, mg/dL	96.9 ± 14.4	98.3 ± 19.8	96.3 ± 12.5	96.3 ± 11.1	96.6 ± 11.3	97.5 ± 11.4	96.4 ± 10.6	<0.001
Creatinine, mg/dL	0.98 ± 0.12	0.95 ± 0.11	0.98 ± 0.11	0.99 ± 0.12	1.01 ± 0.12	1.03 ± 0.12	1.06 ± 0.13	<0.001
eGFR, mL/min/1.73 m^2^	93.1 ± 13.3	95.8 ± 13.8	93.5 ± 13.4	91.8 ± 13.4	90.1 ± 13.5	88.3 ± 13.2	85.7 ± 13.3	<0.001
Total cholesterol, mg/dL	199.9 ± 34.3	193.2 ± 32.8	198.1 ± 33.3	203.7 ± 34.4	208.6 ± 35.3	214.3 ± 36.9	217.5 ± 39.5	<0.001
LDL cholesterol, mg/dL	128.5 ± 31.3	122.0 ± 30.0	127.1 ± 30.5	132.3 ± 31.5	136.4 ± 32.2	140.7 ± 33.2	141.3 ± 33.8	<0.001
HDL cholesterol, mg/dL	52.9 ± 12.8	55.4 ± 13.2	53.5 ± 12.7	51.5 ± 12.2	49.7 ± 11.7	48.6 ± 11.4	47.6 ± 11.0	<0.001
Triglycerides, mg/dL	113 (80–163)	98 (72–139)	108 (78–153)	123 (87–176)	138 (96–199)	155 (106–223)	167 (115–243)	<0.001
PSA, ng/dL	0.82 (0.59–1.13)	0.82 (0.59–1.14)	0.82 (0.60–1.13)	0.81 (0.59–1.13)	0.80 (0.58–1.12)	0.79 (0.57–1.09)	0.80 (0.56–1.11)	<0.001
HOMA-IR	1.29 (0.86–1.90)	1.12 (0.76–1.62)	1.24 (0.83–1.80)	1.40 (0.94–2.04)	1.59 (1.05–2.31)	1.78 (1.19–2.61)	1.83 (1.19–2.73)	<0.001
hsCRP, mg/L	0.5 (0.3–1.0)	0.4 (0.3–0.8)	0.5 (0.3–0.9)	0.6 (0.3–1.1)	0.7 (0.4–1.4)	0.8 (0.5–1.6)	1.0 (0.6–1.9)	<0.001
Total calorie intake, kcal/d ^3^	1636.6 (1306.5–2023.4)	1640.3 (1313.5–2017.0)	1636.9 (1315.4–2012.9)	1636.2 (1302.5–2033.2)	1630.3 (1281.6–2040.9)	1636.1 (1282.2–2066.4)	1608.6 (1262.9–2075.5)	0.448

Data are represented by means ± SD, medians (interquartile range), or percentages. ^1^ ≥20 g/day; ^2^ ≥College graduate; ^3^ Among 71,017 participants with valid estimated energy intake levels (within 3 SD by the log-transformed mean energy intake). BMI, body mass index; BP, blood pressure; eGFR, estimated glomerular filtration rate; HDL, high-density lipoprotein; HEPA, health-enhancing physically active; HOMA-IR, homeostasis model assessment of insulin resistance; hsCRP, high-sensitivity C-reactive protein; LDL, low-density lipoprotein; PSA, prostate-specific antigen.

**Table 2:** Hazard ratios ^1^ (95% CI) of LUTS (>8) by uric acid level.

Uric Acid Level (mg/dL)	Person Years	Incident Case	Incidence Density (per 1000 Person Years)	Age-Adjusted HR ^1^ (95% CI)	Multivariate-Adjusted HR ^1^ (95% CI)	
					Model 1	Model 2
<5.5	96,895.1	3994	41.2	1.00 (reference)	1.00 (reference)	1.00 (reference)
5.5–6.4	125,703.0	4715	37.5	0.98 (0.94–1.02)	0.99 (0.95–1.03)	1.00 (0.96–1.05)
6.5–7.4	88,701.7	3134	35.3	0.96 (0.92–1.01)	0.97 (0.92–1.02)	1.00 (0.95–1.06)
7.5–8.4	35,049.3	1216	34.5	0.98 (0.92–1.04)	0.99 (0.93–1.06)	1.03 (0.96–1.11)
8.5–9.4	9626.9	291	30.2	0.88 (0.78–0.99)	0.90 (0.80–1.02)	0.98 (0.86–1.12)
≥9.5	3006.6	74	24.6	0.73 (0.58–0.92)	0.74 (0.58–0.93)	0.77 (0.59–0.99)
***p* for trend**				0.008	0.039	0.850

^1^ Estimated using a parametric proportional hazard model. BMI, body mass index; eGFR, estimated glomerular filtration rate; HOMA-IR, homeostasis model assessment of insulin resistance; hsCRP, high-sensitivity C-reactive protein; LUTS, lower urinary tract symptoms.

**Table 3:** Hazard ratios ^1^ (95% CI) of LUTS (>8) by the pre-set subgroups.

Subgroup	Uric Acid Level (mg/dL)						*p* for Trend	*p* for Interaction
	<5.5	5.5–6.4	6.5–7.4	7.5–8.4	8.5–9.4	≥9.5		
Age, y								0.261
<50 (n = 95,408)	1.00	0.94 (0.90–0.99)	0.92 (0.87–0.97)	0.91 (0.84–0.98)	0.84 (0.74–0.96)	0.65 (0.50–0.84)	<0.001	
≥50 (n = 5683)	1.00	1.11 (0.94–1.31)	0.90 (0.72–1.11)	1.19 (0.87–1.63)	0.78 (0.39–1.58)		0.687	
Smoking								0.916
Never (n = 61,406)	1.00	1.01 (0.95–1.08)	1.00 (0.93–1.07)	1.03 (0.94–1.14)	0.95 (0.80–1.14)	0.73 (0.52–1.04)	0.619	
Smoker (n = 35,611)	1.00	0.97 (0.90–1.05)	0.97 (0.89–1.05)	0.99 (0.88–1.11)	1.02 (0.83–1.25)	0.82 (0.55–1.21)	0.581	
Alcohol intake, g/day								0.534
<20 (n = 66,029)	1.00	0.98 (0.92–1.04)	0.97 (0.91–1.04)	1.02 (0.93–1.12)	0.90 (0.75–1.07)	0.73 (0.52–1.04)	0.289	
≥20 (n = 33,314)	1.00	1.05 (0.97–1.14)	1.05 (0.96–1.15)	1.07 (0.95–1.20)	1.11 (0.91–1.35)	0.83 (0.57–1.21)	0.353	
HEPA								0.859
No (n = 84,021)	1.00	0.99 (0.94–1.05)	1.00 (0.94–1.06)	1.04 (0.96–1.13)	1.00 (0.87–1.15)	0.76 (0.57–1.00)	0.985	
Yes (n = 16,412)	1.00	1.05 (0.94–1.18)	1.01 (0.89–1.16)	0.99 (0.82–1.19)	0.90 (0.63–1.28)	0.82 (0.44–1.54)	0.621	
BMI (kg/m^2^)								0.182
<25 (n = 61,019)	1.00	1.03 (0.97–1.09)	1.00 (0.94–1.07)	1.02 (0.91–1.13)	1.04 (0.84–1.29)	1.10 (0.71–1.69)	0.689	
≥25 (n = 40,072)	1.00	0.94 (0.86–1.03)	0.98 (0.89–1.07)	1.02 (0.91–1.13)	0.92 (0.78–1.10)	0.64 (0.46–0.88)	0.389	

^1^ Estimated from the parametric proportional hazard model. BMI, body mass index; eGFR, estimated glomerular filtration rate; HOMA-IR, homeostasis model assessment of insulin resistance; hsCRP, high-sensitivity C-reactive protein; LUTS, lower urinary tract symptoms.

## Data Availability

The data presented in this study are available on request from the corresponding author. The data are not publicly available because we do not have permission from the Institutional Review Board to distribute the data.

## Supplementary Materials

The following supporting information can be downloaded at: https://www.mdpi.com/article/10.3390/metabo12070649/s1, Table S1: Hazard ratios (95% CI) of LUTS (>8) by uric acid level after further excluding subjects with diabetes and metabolic syndrome.

## Abbreviations

BMI, body mass index; BP, blood pressure; BPH, benign prostate hyperplasia; CIs, confidence intervals; eGFR, estimated glomerular filtration rate; HDL, high-density lipoprotein; HEPA, health-enhancing physically active; HOMA-IR, homeostasis model of assessment; HRs, hazard ratios; hsCRP, high-sensitivity C-reactive protein; IPSS, International Prostate Symptom Score; LDL, low-density lipoprotein; LUTS, lower urinary tract symptoms; PSA, prostate specific antigen; SUA, serum uric acid; UA, uric acid.

## References

[1] Abrams P., Cardozo L., Fall M., Griffiths D., Rosier P., Ulmsten U., van Kerrebroeck P., Victor A., Wein A. (2002). The standardisation of terminology of lower urinary tract function: Report from the Standardisation Sub-committee of the International Continence Society. Am. J. Obstet. Gynecol..

[2] Coyne K.S., Sexton C.C., Thompson C.L., Milsom I., Irwin D., Kopp Z.S., Chapple C.R., Kaplan S., Tubaro A., Aiyer L.P. (2009). The prevalence of lower urinary tract symptoms (LUTS) in the USA, the UK and Sweden: Results from the Epidemiology of LUTS (EpiLUTS) study. BJU Int..

[3] Yoo T.K., Lee K.S., Sumarsono B., Kim S.T., Kim H.J., Lee H.C., Kim S.H. (2018). The prevalence of lower urinary tract symptoms in population aged 40 years or over, in South Korea. Investig. Clin. Urol..

[4] Cambronero Santos J., Errando Smet C. (2016). Prevalence of storage lower urinary tract symptoms in male patients attending Spanish urology office. Urinary urgency as predictor of quality of life. Actas Urol. Esp..

[5] Lee K.S., Yoo T.K., Liao L., Wang J., Chuang Y.C., Liu S.P., Chu R., Sumarsono B. (2017). Association of lower urinary tract symptoms and OAB severity with quality of life and mental health in China, Taiwan and South Korea: Results from a cross-sectional, population-based study. BMC Urol..

[6] Lepor H. (2005). Pathophysiology of lower urinary tract symptoms in the aging male population. Rev. Urol..

[7] Lee C.L., Kuo H.C. (2017). Current consensus and controversy on the diagnosis of male lower urinary tract symptoms/benign prostatic hyperplasia. Tzu-Chi Med. J..

[8] De Nunzio C., Roehrborn C.G., Andersson K.E., McVary K.T. (2017). Erectile Dysfunction and Lower Urinary Tract Symptoms. Eur. Urol. Focus.

[9] Li Z., Huang W., Wang X., Zhang Y. (2018). The relationship between lower urinary tract symptoms and irritable bowel syndrome: A meta-analysis of cross-sectional studies. Minerva Urol. Nefrol..

[10] Barbosa J.A., Muracca E., Nakano E., Paranhos M., Natalino R., Cordeiro P., Srougi M., Antunes A.A. (2013). Risk factors for male lower urinary tract symptoms: The role of metabolic syndrome and androgenetic alopecia in a Latin American population. Urology.

[11] Lee J.H., Park Y.W., Lee S.W. (2019). The Relationships between Thyroid Hormone Levels and Lower Urinary Tract Symptoms/Benign Prostatic Hyperplasia. World J. Mens Health.

[12] Sanford M.T., Rodriguez L.V. (2017). The role of environmental stress on lower urinary tract symptoms. Curr. Opin. Urol..

[13] De Nunzio C., Presicce F., Tubaro A. (2016). Inflammatory mediators in the development and progression of benign prostatic hyperplasia. Nat. Rev. Urol..

[14] Kukko V., Kaipia A., Talala K., Taari K., Tammela T.L.J., Auvinen A., Murtola T.J. (2018). Allopurinol and risk of benign prostatic hyperplasia in a Finnish population-based cohort. Prostate Cancer Prostatic Dis..

[15] Sangkop F., Singh G., Rodrigues E., Gold E., Bahn A. (2016). Uric acid: A modulator of prostate cells and activin sensitivity. Mol. Cell Biochem..

[16] Kanbara A., Hakoda M., Seyama I. (2010). Urine alkalization facilitates uric acid excretion. Nutr. J..

[17] Franceschi C., Garagnani P., Parini P., Giuliani C., Santoro A. (2018). Inflammaging: A new immune-metabolic viewpoint for age-related diseases. Nat. Rev. Endocrinol..

[18] Persson B.E., Ronquist G. (1996). Evidence for a mechanistic association between nonbacterial prostatitis and levels of urate and creatinine in expressed prostatic secretion. J. Urol..

[19] Doblado M., Moley K.H. (2009). Facilitative glucose transporter 9, a unique hexose and urate transporter. Am. J. Physiol. Endocrinol. Metab..

[20] Das Gupta E., Sakthiswary R., Lee S.L., Wong S.F., Hussein H., Gun S.C. (2018). Clinical significance of SLC2A9/GLUT9 rs11722228 polymorphisms in gout. Int. J. Rheum. Dis..

[21] Uemura N., Murakami R., Chiba Y., Yanase K., Fujihara R., Mashima M., Matsumoto K., Kawauchi M., Shirakami G., Ueno M. (2017). Immunoreactivity of urate transporters, GLUT9 and URAT1, is located in epithelial cells of the choroid plexus of human brains. Neurosci. Lett..

[22] Song J.U., Hwang J., Ahn J.K. (2017). Serum uric acid is positively associated with pulmonary function in Korean health screening examinees. Mod. Rheumatol..

[23] Hwang J., Hwang J.H., Ryu S., Ahn J.K. (2019). Higher serum uric acid is associated with higher lumbar spine bone mineral density in male health-screening examinees: A cross-sectional study. J. Bone Miner. Metab..

[24] Hwang J., Hwang J.H., Chung S.M., Kwon M.J., Ahn J.K. (2018). Association between serum uric acid and arterial stiffness in a low-risk, middle-aged, large Korean population: A cross-sectional study. Medicine.

[25] Oh J.Y., Yang Y.J., Kim B.S., Kang J.H. (2007). Validity and Reliability of Korean Version of International Physical Activity Questionnaire (IPAQ) Short Form. Korean J. Fam. Med..

[26] Kim M.K., Lee W.Y., Kang J.H., Kang J.H., Kim B.T., Kim S.M., Kim E.M., Suh S.H., Shin H.J., Lee K.R. (2014). 2014 clinical practice guidelines for overweight and obesity in Korea. Endocrinol. Metab..

[27] Park H.J., Park C.H., Chang Y., Ryu S. (2018). Sitting time, physical activity and the risk of lower urinary tract symptoms: A cohort study. BJU Int..

[28] Levey A.S., Stevens L.A., Schmid C.H., Zhang Y.L., Castro A.F., Feldman H.I., Kusek J.W., Eggers P., Van Lente F., Greene T. (2009). A new equation to estimate glomerular filtration rate. Ann. Intern. Med..

[29] Kim J.H., Kim S.C. (2007). Validation of Korean Version of International Prostate Symptom Score: A Comparison of Physician versus Self-administration. Korean J. Androl..

