# Supplementary material for: Higher Levels of Serum Uric Acid Have a Significant Association with Lower Incidence of Lower Urinary Tract Symptoms in Healthy Korean Men
Source: Metabolites. 2022 Jul 14;12(7):649. doi: 10.3390/metabo12070649 (PMC9322789; doi:10.3390/metabo12070649)
Supplement: Supplementary file 1 [file metabolites-12-00649-s001.zip › metabolites-1799349-supplementary.pdf]

Supplementary Table S1. Hazard ratios[1] (95% CI) of LUTS (> 8) by uric acid level after further excluding subjects with diabetes and metabolic syndrome.

Uric Acid Level (mg/dL)	Multivariate-Adjusted HR[1] (95% CI)	
	After further excluding 3637 participants with diabetes (n = 97,454)	After further excluding 13148 participants with metabolic syndrome (n = 87,943)
**<5.5**	1.00 (reference)	1.00 (reference)
**5.5–6.4**	0.99 (0.95–1.03)	0.99 (0.95–1.04)
**6.5–7.4**	0.97 (0.92–1.02)	0.97 (0.92–1.02)
**7.5–8.4**	1.02 (0.94–1.07)	1.03 (0.95–1.10)
**8.5–9.4**	0.89 (0.78–1.01)	0.88 (0.76–1.02)
**≥9.5**	0.73 (0.58–0.93)	0.75 (0.56–0.99)
**p for trend**	0.051	0.174

[1]Estimated from parametric proportional hazard model.

The multivariable model was adjusted for age, center, year of a screening exam, smoking status, alcohol intake, physical activity, educational level, total calorie intake, BMI, history of diabetes, history of hypertension, eGFR, total cholesterol, HOMA-IR, and hsCRP. Diabetes was defined as a fasting glucose concentration ≥ 126 mg/dL, glycated hemoglobin concentration ≥ 6.5%, or currently taking antidiabetic medications or insulin. Metabolic syndrome was determined by having three or more components among five components: triglyceride ≥ 150 mg/dL; high-density lipoprotein < 40 mg/dL; systolic blood pressure ≥ 130 mmHg and/or diastolic blood pressure ≥ 85 mmHg or use of antihypertensive medication; fasting glucose ≥ 100 mg/dL or use of anti-diabetic medication, and abdominal obesity. BMI, body mass index; eGFR, estimated glomerular filtration rate; HOMA-IR, homeostasis model assessment of insulin resistance; hsCRP, high-sensitivity C-reactive protein; LUTS, lower urinary tract symptoms.
